# High Birefringence D-Shaped Germanium-Doped Photonic Crystal Fiber Sensor

**DOI:** 10.3390/mi13060826

**Published:** 2022-05-25

**Authors:** Qianhe Zhao, Jin Liu, Haima Yang, Haishan Liu, Guohui Zeng, Bo Huang

**Affiliations:** 1 School of Electronic and Electrical Engineering, Shanghai University of Engineering Science, Shanghai 201620, China; m020219328@sues.edu.cn (Q.Z.); hithsh@163.com (H.L.); zenggh@sues.edu.cn (G.Z.); huangbosues@sues.edu.cn (B.H.); 2 School of Optical-Electrical and Computer Engineering, University of Shanghai for Science and Technology, Shanghai 200093, China; snowyhm@sina.com

**Keywords:** fiber optic sensor, surface plasmon resonance, finite element method, birefringence

## Abstract

In this work, a surface plasmon resonance (SPR) sensor based on a D-shaped germanium-doped photonic crystal fiber (PCF) is proposed. The finite element method (FEM) is introduced to analyze the structure parameters, such as germanium-doped concentration, lattice pitch, and air hole size. In addition, the coupling properties and birefringence properties of PCF are also studied. The computer simulation results indicate that two different surface plasmon polariton (SPP) coupling modes are produced on the polished surface, covered with metal film, when the analyte refractive index (RI) is 1.34. Then, with the increase of the RI, the incompleteness of one of the coupling modes will be transformed into the complete coupling. The effect of germanium concentration on the birefringence is also analyzed. It has an optimal wavelength sensitivity of 5600 nm/RIU when the RI is 1.37. This sensor exhibits a maximum birefringence of 1.06 × 10^−2^ and a resolution of 1.78 × 10^−5^ RIU with high linearity.

## 1. Introduction

Surface plasmon resonance (SPR) occurs when the evanescent wave formed by light at the interface of the metal–dielectric resonates with electrons. Optical fiber was originally widely used in the field of communications. It was later discovered that the periodic arrangement of air holes inside the PCF gives fiber designable and controllable polarization characteristics, dispersion characteristics, etc., so it is also ideal for SPR sensors [1,2]. Compared with conventional prism-based SPR sensors, the PCF-SPR sensor has the advantages of high sensitivity, fast response time, and flexible structure, and it is also superior in real-time sensing and remote sensing [3,4,5].

However, in the conventional PCF-SPR sensors, researchers usually fill the inside of the fiber with the analyte, and the metal layer is also coated inside the air hole, which is tedious and difficult in practice, so some researchers have focused their research on a D-shaped PCF [6,7,8,9,10]. A D-shaped PCF changes its structure by side-polishing the traditional fiber, making it easier for metal to deposit on the polished surface, and the D-shaped PCF can solve the difficult problem of SPR phase matching. Cheng Zhou reported a PCF-SPR sensor that was based on a self-referencing channel, and the spectral sensitivity of this sensor was only dependent on the thickness of the metal. With a silver film thickness of 50 nm, the maximum spectral sensitivity of the sensor was 3191.43 nm/RIU [11]. J. N. Dashet studied a D-shaped PCF-SPR biosensor with a silver-graphene layer. Graphene facilitates the absorption of biomolecules and prevents the oxidation of metals. The sensor has a spectral sensitivity of 3700 nm/RIU [12].

In some conventional PCF fibers, the difference in propagation constants between the x and y polarized of the fundamental mode is small because the core and cladding are symmetrical, thus making the birefringence characteristics not obvious [13]. Since PCF sensors with high birefringence have the advantage of low-crosstalk, high-resolution, and maintaining the polarization state of the light, researchers have often resorted to breaking the symmetry of the fiber structure to enhance the birefringence properties [14,15]. However, this method usually makes the arrangement of the air holes not close enough, which causes the core energy to leak into the cladding.

To solve these problems, a high birefringence D-shaped germanium-doped PCF-SPR sensor with a high sensitivity is presented. The operation of side polishing of PCF and the installation of an external sensitive layer on the polished surface reduces the preparation difficulty and enables real-time inspection. The symmetry of the fiber structure is broken by the introduction of four small elliptical air holes in the D-shaped PCF core. At the same time, the light confinement of the core is improved by doping the core with germanium. The asymmetric air-hole structure and the germanium-doped core improve the birefringence of the PCF, while achieving high sensitivity and high linearity. By analyzing the germanium-doped concentration, the relationship between germanium ions and birefringence was verified. The simulation results showed that the sensor proposed in this paper cannot only achieve high birefringence but also makes the PCF-SPR have better sensitivity within a certain refractive index (RI) range.

## 2. Sensing Principle and Experimental Setup

**Figure 1:**
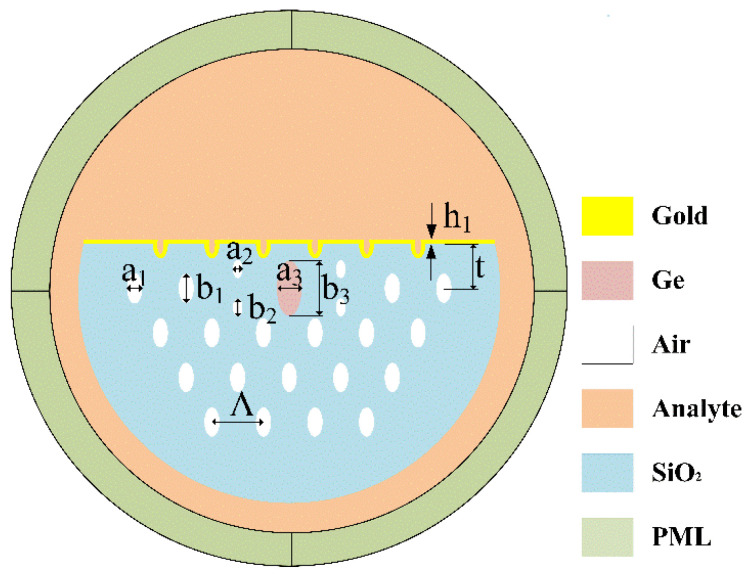
The D-shaped PCF sensor model proposed in this paper.

Figure 1 shows the schematic diagram of the germanium-doped PCF-SPR sensor. There is an oval lotus pink germanium-doped area in the center of the PCF. The cladding area of the PCF is hexagonal air holes (white), where the air holes are composed of two ellipses of different sizes. The design of small air holes and elliptical cores enhances the birefringence of the PCF-SPR, and the increased birefringence facilitates the coupling of the incident light to a specific direction, thus enhancing the coupling between the fundamental mode and the SPP mode [16,17]. In the cladding area, the semi-major and semi-minor axes of the large air holes are $b_{1}$ = 0.5 μm, $a_{1}$ = 0.25 μm, respectively, and the lattice pitch is ∧ = 2 μm. The semi-major and semi-minor axes of the small air holes are $b_{2}$ = 0.3 μm, $a_{2}$ = 0.15 μm, respectively. In the core region, the semi-major and semi-minor axes of the elliptical germanium-doped region are $b_{3}$ = 0.88 μm, $a_{3}$ = 0.4 μm. The radius of the photonic crystal fiber is r=3.5∧. A gold film (yellow) of thickness $h_{1}$ = 50 nm is deposited on the polished surface of the D-shaped PCF, which enables the excitation of surface plasma polaritons (SPP). t=sqrt(3)∧/2 is the distance from the plane of polishing to the fiber core; analyte liquid can flow over the top of the cross-section, and the RI of the analyte $n_{a}=1.34$. The D-shaped PCF-SPR can be manufactured by the side polishing method [18]. In the construction of this paper, the fiber is made of silica, whose RI is established by the Sellmeier formula [19]:

(1) $$n_{SiO_{2}}=1+\frac{B_{1}\lambda }{\lambda -A_{1}}+\frac{B_{2}\lambda }{\lambda -A_{2}}+\frac{B_{3}\lambda }{\lambda -A_{3}}$$

where *λ* is the working wavelength; $A_{1}$, $A_{2}$, $A_{3}$, $B_{1}$, $B_{2}$, and $B_{3}$ are called Sellmeier constants.

The concentration of germanium is e; then, the RI of the germanium-doped region of the ellipse is:

(2) $$n_{Ge}=n_{SiO_{2}}(1+e)$$

In PCF, a mode analysis is performed on the propagation cross-section of the incident light in the core. Using the FEM, we can obtain the effective refractive index ($n_{eff}$) of the fundamental mode, while the birefringence of the PCF is defined by the difference between the real part of the effective refractive index (Re(neff)) of the x- and the y-polarized:

(3) $$B=\left|n_{eff}^{x}-n_{eff}^{y}\right|$$

In the formula,$n_{eff}^{x}$, $n_{eff}^{y}$ is Re(neff) of the x- and y-polarized of the fundamental mode, respectively. When the birefringence is large enough, the two orthogonal polarization modes of the incident light can be effectively separated, thus reducing the polarization state unmeasurability during fiber transmission and ensuring the stability of the PCF-SPR sensor [20].

**Figure 2:**
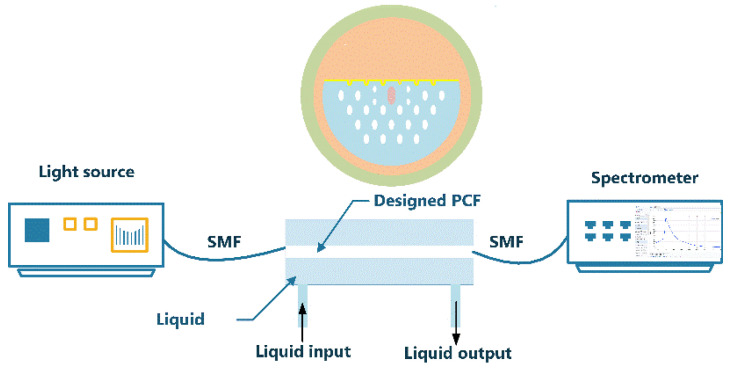
The experimental setup of the sensor.

The confinement loss of the PCF at different wavelengths is given by the following equation [21]:

(4) αloss=8.686×k0×Im(neff)×104

where $k_{0}=\frac{2\pi }{\lambda }$ is the vacuum wave number. From this, we can know that the confinement loss is proportional to the imaginary part of the effective refractive index (Im(neff)). To obtain accurate calculation results, a Perfect Matching Layer (PML) is added along the exterior of the model to absorb all waves from the simulation area [22]. The simulation process is completed by the multi-physics simulation software COMSOL MultiPhysical. The experimental device is to place the PCF-SPR sensor between two single-mode fibers (SMF). The light from the white light source is transmitted through the designed PCF, and finally, the spectral response was measured using an optical spectrum analyzer. Figure 2 shows the experimental set-up of the sensor.

## 3. Results and Discussion

### 3.1. Phase Matching

To investigate the sensing properties of the PCF-SPR sensor, the phase matching and loss matching of this PCF need to be analyzed. Normally, the resonant coupling phenomenon between the fundamental and SPP modes is numerically represented by the intersection of their dispersion relations, which also implies that the propagation constants of the two modes are equal [23,24]. 

**Figure 3:**
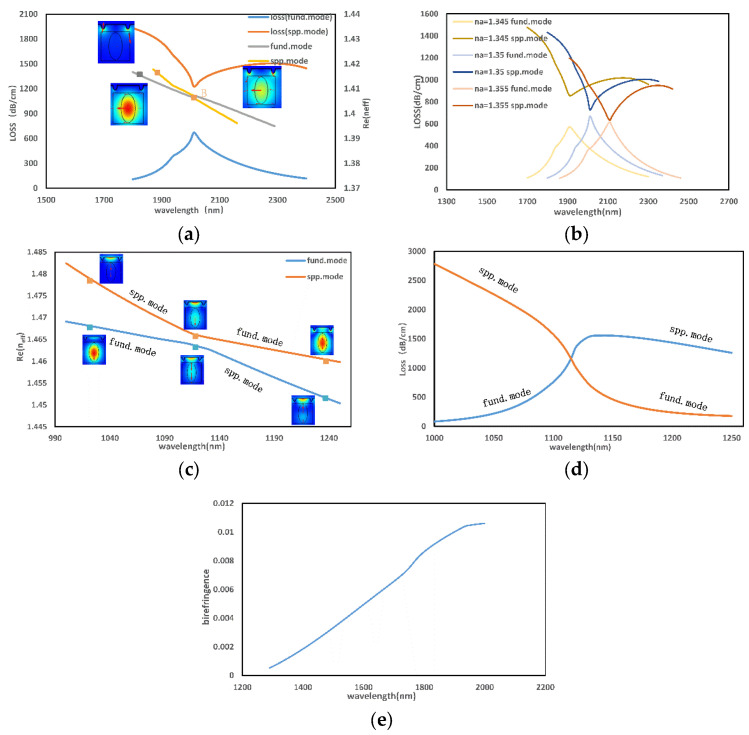
(**a**) is the electric field distribution and dispersion characteristics of the x-polarized of the fund.mode when $n_{a}=1.34$; (**b**) is the loss spectrum of x-polarized when $n_{a}=$ 1.345–1.355; (**c**,**d**) are the Re(neff) curve and loss spectrum of the y-polarized of the fund.mode, respectively; (**e**) is the birefringence of PCF when $n_{a}=1.34$.

Figure 3a,d show the loss spectra of the x-polarized and y-polarized of the fundamental mode. It can be observed that the resonance peaks appear in the near-infrared band, and both polarization states of the fundamental mode exhibit single resonance properties. The following is an analysis of the x-polarized: as can be seen in Figure 3a, the phase-matching condition between the x-polarized and SPP modes is satisfied at point B, and the confinement loss reaches both peak and trough values. However, the loss matching condition is not met. The propagation constant β of the mode field can be derived from the mode coupling theory of PCF as:

(5) $$\beta_{\pm }=\beta_{ave}\pm \sqrt{\delta^{2}+k^{2}}$$

of which:

(6) $$\beta_{ave}=\frac{\beta_{1}+\beta_{2}}{2}$$

(7) $$\delta =\frac{\beta_{1}-\beta_{2}}{2}$$

Furthermore, $\beta {\;}_{1}$, $\beta {\;}_{2}$ are the propagation constants of the fiber core fundamental mode and SPP mode, respectively. $\beta_{1}$, $\beta_{2}$ and δ are complex numbers. For the leaky mode, when $\delta_{i}>k$, the propagation constants of the two modes have the same real part and different imaginary part, and incomplete coupling will occur at this time.

According to Figure 3a, it can be judged that the coupling of the x-polarized and the SPP mode is incompletely coupled when $n_{a}=1.$4. From the subsequent simulation, it is found that as $n_{a}$ increases, the peak of the confinement loss of the x-polarized will increase, while the valley of the confinement loss of the SPP mode will decrease, thus resulting in a gradual decrease of the difference between the two loss values. As shown in Figure 3b, the difference in the loss value is 0, and the loss matching conditions of the two modes can be satisfied when $n_{a}\geq 1.355$.

As shown in Figure 3c, an anti-crossing effect occurs during the coupling between the y-polarized of the fundamental mode and the SPP mode. As the wavelength increases near the anti-crossing point, the loss curves of the SPP mode and y-polarized intersect and the electric field distribution is almost identical. After this point, the energy distribution of the two modes shows an opposite trend. The energy transition can also be demonstrated by the change in the loss value in Figure 3d. The intersection of the loss spectra proves that the $Im\left(n_{eff}\right)$ of the two modes are the same, so the SPP mode and the y-polarized reach the coupling condition. 

From the above discussion, it can be concluded that in the whole wavelength range, the loss spectrum of x-polarized is sharper and more sensitive to the change of RI. According to Figure 3e, as the wavelength increases, the Re(neff) difference between the two polarization modes increases, which means that the effect of birefringence becomes more prominent. The birefringence will cause a strong coupling of the polarization mode with the fundamental mode, which enhances the sensitivity of the sensor [15]. The resonant wavelength of the x-polarized lies at a much larger wavelength, so the following will focus on the sensing properties of the x-polarized of the fundamental mode.

### 3.2. Optimization of Structural Parameters

**Figure 4:**
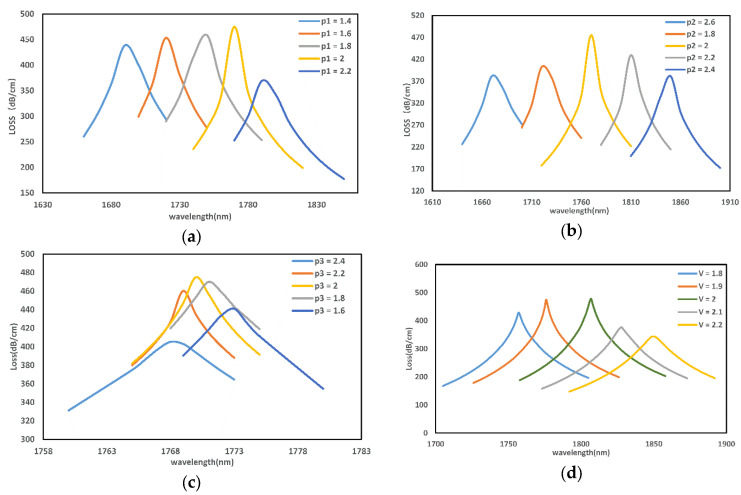
Loss spectra under different cladding air holes (**a**), germanium-doped regions (**b**), air holes in the fiber core region (**c**), and lattice constant (**d**).

Next, the influence of structural parameters on resonance spectra will be systematically studied. Figure 4 shows the loss spectrum when the ellipticity and lattice constant Λ of the elliptical air hole is changed, where $p_{1}=b_{1}/a_{1},p_{2}=b_{2}/a_{2},p_{3}=b_{3}/a_{3}$ and $n_{a}=1.355$.

Figure 4a shows that the resonant wavelength becomes larger as p1 increases. During $p_{1}=$ 1.4–2, the coupling is gradually enhanced as the air holes increase, bringing the metal film closer to the fiber core. When $p_{1}=$ 2–2.2, as $\;p_{1}$ increases, the core confinement capacity starts to increase, and the confinement loss gradually decreases. 

Figure 4b shows that with the increase of $p_{2}$, the ellipticity of the germanium-doped region increases, and the loss curve shows a trend of increasing and then decreasing. The resonance peak of the sensor is most prominent at $p_{2}=2$. 

It is evident from Figure 4c that as the ellipticity of the smallest air hole increases, the loss spectrum produces a blue shift. When $p_{3}=2$, the loss reaches the highest value, indicating that the ellipticity at this value has the best effect on promoting phase matching.

It can be inferred from Figure 4d that as the lattice constant of the PCF increases, the $n_{eff}$ of the fundamental mode will decrease, and at this time, the $n_{eff}$ of the SPP mode will increase, so the loss spectrum also appears as a redshift.

**Figure 5:**
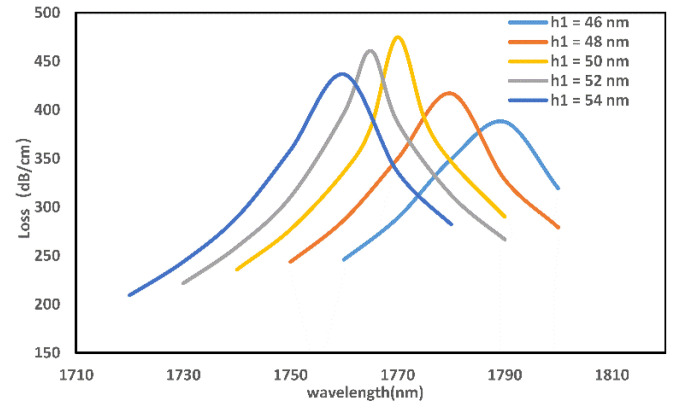
Sensor loss curve for different gold film thicknesses.

Covering the surface of the fiber with a metal film is necessary to achieve the SPR phenomenon. Figure 5 indicates the loss peak of the sensor for a gold film of thickness of $h_{1}=$ 46 nm–50 nm. As shown in the figure, as the thickness increases, the loss spectrum exhibits a blue shift. As $h_{1}$ gradually changes from 54 nm to 50 nm, the resonance intensity increases due to the thinning of the gold film, reaching the highest loss value at $h_{1}=50\;\mathrm{nm}$. Therefore, 50 nm may be an optimum gold film thickness.

### 3.3. Analysis of Birefringence Effect

Because the structure of the traditional single-mode fiber is symmetrical, the propagation constants of the two polarization directions of the fundamental mode are equal. However, the actual manufacturing does not guarantee the complete symmetry of the internal structure of the fiber, which can cause the change of the two polarization modes and thus the birefringence phenomenon [25]. Therefore, the characteristic of high birefringence also has an important influence and significance in the research. In this work, two methods are used to improve the birefringence effect of the sensor: doping with germanium and breaking the symmetry of the air holes in the PCF.

**Figure 6:**
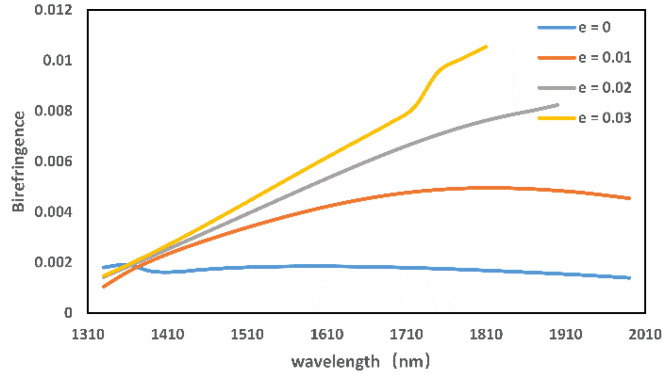
Effect of different germanium concentrations on birefringence.

When the core region is doped with germanium, the core RI increases. However, the RI of the cladding remains unchanged, thus enhancing the optical confinement of the fiber and allowing the mode field to become more concentrated. Figure 6 shows that the birefringence is up to 1.06 × 10^−2^ at e=0.03. It can be seen from the figure that the fiber with germanium-doped has a higher birefringence than ordinary fibers without germanium-doped, and the effect of birefringence is better with increasing germanium content within a certain range. At the same time, because the symmetry of the fiber structure is broken, a part of the mode field is distributed in the cladding when the incident wavelength is long. As a result, the birefringence effect becomes more pronounced as the wavelength increases.

### 3.4. Sensing Performance

**Figure 7:**
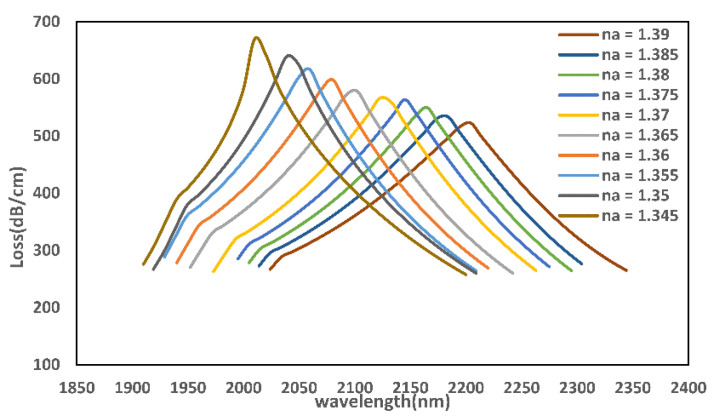
Loss curve of the sensor at different $n_{a}$.

The sensing properties of the PCF sensor are discussed next. Figure 7 presents the loss peak of the sensor suggested in this paper for different analyte RIs. When $n_{a}$ increases, the resonance wavelength will appear as a redshift. When $n_{a}\;\geq \;1.355$, an anti-crossing effect occurs, and the coupling phenomenon between modes varies from incomplete to complete coupling.

The sensitivity of the RI sensor is denoted by S, and its unit is nm/RIU. The sensitivity equation is expressed as follows:

(8) $$S_{\lambda }=\frac{\Delta \lambda_{peak}}{\Delta n_{a}}$$

where $\Delta \lambda_{peak}$ is the variation of the corresponding resonance wavelength when the RI value changes by $\Delta n_{a}$.

**Figure 8:**
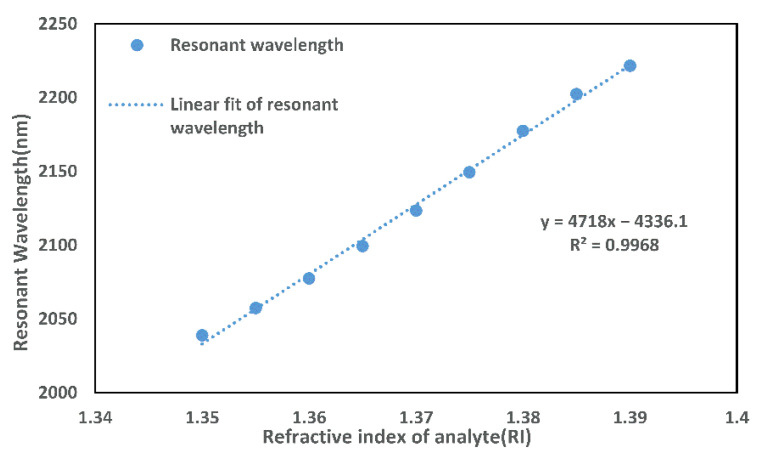
Fitting curve of resonance wavelength with RI.

Figure 8 shows the linear relationship between resonance wavelength and RI for $n_{a}=$ 1.345–1.39. The formula in the figure is the result of the linear fitting. The fitted line has $R^{2}=0.9968$. The larger the R-Square, the better the linear fit of the curve, which demonstrates that the sensor has a low non-linear error. The average wavelength sensitivity of this PCF-SPR sensor is 4718 nm/RIU when $n_{a}=$ 1.35–1.39. When $n_{a}=1.37$, it has the highest sensitivity of 5600 nm/RIU.

The definition of the resolution of the RI is:

(9) $$R=\frac{\Delta n_{a}\Delta \lambda_{\min}}{\Delta \lambda_{peak}}$$

where $\Delta \lambda_{\min}$ is the assumed minimum spectral resolution of 0.1 nm. The maximum resolution of this structure is 1.78 × 10^−5^ RIU. The performance of the sensor proposed in this paper is compared with other PCF-SPR sensors in Table 1. The table shows that the sensor proposed in this paper has certain advantages in terms of sensitivity, resolution, and linearity.

## 4. Conclusions

The proposed sensor not only increases the birefringent of the PCF by adding germanium and designing air holes, but it also reduces the beam transmission distance by polishing the D-shaped PCF, thus reducing the difficulty of phase matching of the SPR sensor and improving the sensitivity of the sensor. The simulation results show that the presented PCF sensor has a high sensitivity, high birefringence, and high linearity within $n_{a}=$ 1.35–1.39, making the sensor competitive for liquid and gas detection.

## Figures and Tables

**Table 1:** Comparison of Various Fiber-SPR Sensor Performances.

Sensor Features	RI Range	Average Sensitivity (nm/RIU)	Resolution (RIU)	Linearity
D-Shaped Photonic Crystal Fiber With Split Cladding Air Holes [26]	1.335–1.365	2331.9	—	—
Multi-Channel PCF-SPR Sensor [27]	1.33–1.37	3083	4 × 10^−5^	0.9832
Sensors Based on Self-Reference Channel	1.33–1.38	3191.43	—	—
H-Shaped Photonic Crystal Fibers [28]	1.33–1.36	2770	3.61 × 10^−5^	—
Ultra-Wide Detection Range [29]	1.29–1.49	3703.64	—	0.99236
This work	1.35–1.39	4718	1.78 × 10^−5^	0.9968

## Data Availability

No new data were created or analyzed in this study. Data sharing is not applicable to this article.
